# A roadmap to using randomization in clinical trials

**DOI:** 10.1186/s12874-021-01303-z

**Published:** 2021-08-16

**Authors:** Vance W. Berger, Louis Joseph Bour, Kerstine Carter, Jonathan J. Chipman, Colin C. Everett, Nicole Heussen, Catherine Hewitt, Ralf-Dieter Hilgers, Yuqun Abigail Luo, Jone Renteria, Yevgen Ryeznik, Oleksandr Sverdlov, Diane Uschner, for the Randomization Innovative Design Scientific Working Group, Robert A Beckman

**Affiliations:** 1 National Institutes of Health, Bethesda, MD USA; 2 Boehringer Ingelheim Pharma GmbH & Co. KG, Biberach, Germany; 3 Boehringer-Ingelheim Pharmaceuticals Inc, Ridgefield, CT USA; 4 Population Health Sciences, University of Utah School of Medicine, Salt Lake City UT, USA; 5 Cancer Biostatistics, University of Utah Huntsman Cancer Institute, Salt Lake City UT, USA; 6 Clinical Trials Research Unit, University of Leeds, Leeds, UK; 7 RWTH Aachen University, Aachen, Germany; 8 Medical School, Sigmund Freud University, Vienna, Austria; 9 York Trials Unit, Department of Health Sciences, University of York, York, UK; 10 Food and Drug Administration, Silver Spring, MD USA; 11 Open University of Catalonia (UOC) and the University of Barcelona (UB), Barcelona, Spain; 12 Department of Human Development and Quantitative Methodology, University of Maryland, College Park, MD USA; 13 BioPharma Early Biometrics & Statistical Innovations, Data Science & AI, R&D BioPharmaceuticals, AstraZeneca, Gothenburg, Sweden; 14 Early Development Analytics, Novartis Pharmaceuticals Corporation, NJ East Hanover, USA; 15 Biostatistics Center & Department of Biostatistics and Bioinformatics, George Washington University, DC Washington, USA

**Keywords:** Balance, Randomization-based test, Restricted randomization design, Validity

## Abstract

**Background:**

Randomization is the foundation of any clinical trial involving treatment comparison. It helps mitigate selection bias, promotes similarity of treatment groups with respect to important known and unknown confounders, and contributes to the validity of statistical tests. Various restricted randomization procedures with different probabilistic structures and different statistical properties are available. The goal of this paper is to present a systematic roadmap for the choice and application of a restricted randomization procedure in a clinical trial.

**Methods:**

We survey available restricted randomization procedures for sequential allocation of subjects in a randomized, comparative, parallel group clinical trial with equal (1:1) allocation. We explore statistical properties of these procedures, including balance/randomness tradeoff, type I error rate and power. We perform head-to-head comparisons of different procedures through simulation under various experimental scenarios, including cases when common model assumptions are violated. We also provide some real-life clinical trial examples to illustrate the thinking process for selecting a randomization procedure for implementation in practice.

**Results:**

Restricted randomization procedures targeting 1:1 allocation vary in the degree of balance/randomness they induce, and more importantly, they vary in terms of validity and efficiency of statistical inference when common model assumptions are violated (e.g. when outcomes are affected by a linear time trend; measurement error distribution is misspecified; or selection bias is introduced in the experiment). Some procedures are more robust than others. Covariate-adjusted analysis may be essential to ensure validity of the results. Special considerations are required when selecting a randomization procedure for a clinical trial with very small sample size.

**Conclusions:**

The choice of randomization design, data analytic technique (parametric or nonparametric), and analysis strategy (randomization-based or population model-based) are all very important considerations. Randomization-based tests are robust and valid alternatives to likelihood-based tests and should be considered more frequently by clinical investigators.

**Supplementary Information:**

The online version contains supplementary material available at 10.1186/s12874-021-01303-z.

## Background

Various research designs can be used to acquire scientific medical evidence. The randomized controlled trial (RCT) has been recognized as the most credible research design for investigations of the clinical effectiveness of new medical interventions [1, 2]. Evidence from RCTs is widely used as a basis for submissions of regulatory dossiers in request of marketing authorization for new drugs, biologics, and medical devices. Three important methodological pillars of the modern RCT include blinding (masking), randomization, and the use of control group [3].

While RCTs provide the highest standard of clinical evidence, they are laborious and costly, in terms of both time and material resources. There are alternative designs, such as observational studies with either a cohort or case–control design, and studies using real world evidence (RWE). When properly designed and implemented, observational studies can sometimes produce similar estimates of treatment effects to those found in RCTs, and furthermore, such studies may be viable alternatives to RCTs in many settings where RCTs are not feasible and/or not ethical. In the era of big data, the sources of clinically relevant data are increasingly rich and include electronic health records, data collected from wearable devices, health claims data, etc. Big data creates vast opportunities for development and implementation of novel frameworks for comparative effectiveness research [4], and RWE studies nowadays can be implemented rapidly and relatively easily. But how credible are the results from such studies?

In 1980, D. P. Byar issued warnings and highlighted potential methodological problems with comparison of treatment effects using observational databases [5]. Many of these issues still persist and actually become paramount during the ongoing COVID-19 pandemic when global scientific efforts are made to find safe and efficacious vaccines and treatments as soon as possible. While some challenges pertinent to RWE studies are related to the choice of proper research methodology, some additional challenges arise from increasing requirements of health authorities and editorial boards of medical journals for the investigators to present evidence of transparency and reproducibility of their conducted clinical research. Recently, two top medical journals, the New England Journal of Medicine and the Lancet, retracted two COVID-19 studies that relied on observational registry data [6, 7]. The retractions were made at the request of the authors who were unable to ensure reproducibility of the results [8]. Undoubtedly, such cases are harmful in many ways. The already approved drugs may be wrongly labeled as “toxic” or “inefficacious”, and the reputation of the drug developers could be blemished or destroyed. Therefore, the highest standards for design, conduct, analysis, and reporting of clinical research studies are now needed more than ever. When treatment effects are modest, yet still clinically meaningful, a double-blind, randomized, controlled clinical trial design helps detect these differences while adjusting for possible confounders and adequately controlling the chances of both false positive and false negative findings.

Randomization in clinical trials has been an important area of methodological research in biostatistics since the pioneering work of A. Bradford Hill in the 1940’s and the first published randomized trial comparing streptomycin with a non-treatment control [9]. Statisticians around the world have worked intensively to elaborate the value, properties, and refinement of randomization procedures with an incredible record of publication [10]. In particular, a recent EU-funded project (www.IDeAl.rwth-aachen.de) on innovative design and analysis of small population trials has “randomization” as one work package. In 2020, a group of trial statisticians around the world from different sectors formed a subgroup of the Drug Information Association (DIA) Innovative Designs Scientific Working Group (IDSWG) to raise awareness of the full potential of randomization to improve trial quality, validity and rigor (https://randomization-working-group.rwth-aachen.de/).

The aims of the current paper are three-fold. First, we describe major recent methodological advances in randomization, including different restricted randomization designs that have superior statistical properties compared to some widely used procedures such as permuted block designs. Second, we discuss different types of experimental biases in clinical trials and explain how a carefully chosen randomization design can mitigate risks of these biases. Third, we provide a systematic roadmap for evaluating different restricted randomization procedures and selecting an “optimal” one for a particular trial. We also showcase application of these ideas through several real life RCT examples.

The target audience for this paper would be clinical investigators and biostatisticians who are tasked with the design, conduct, analysis, and interpretation of clinical trial results, as well as regulatory and scientific/medical journal reviewers. Recognizing the breadth of the concept of randomization, in this paper we focus on a randomized, comparative, parallel group clinical trial design with equal (1:1) allocation, which is typically implemented using some restricted randomization procedure, possibly stratified by some important baseline prognostic factor(s) and/or study center. Some of our findings and recommendations are generalizable to more complex clinical trial settings. We shall highlight these generalizations and outline additional important considerations that fall outside the scope of the current paper.

The paper is organized as follows. The “Methods” section provides some general background on the methodology of randomization in clinical trials, describes existing restricted randomization procedures, and discusses some important criteria for comparison of these procedures in practice. In the “Results” section, we present our findings from four simulation studies that illustrate the thinking process when evaluating different randomization design options at the study planning stage. The “Conclusions” section summarizes the key findings and important considerations on restricted randomization procedures, and it also highlights some extensions and further topics on randomization in clinical trials.

## Methods

### What is randomization and what are its virtues in clinical trials?

Randomization is an essential component of an experimental design in general and clinical trials in particular. Its history goes back to R. A. Fisher and his classic book “The Design of Experiments” [11]. Implementation of randomization in clinical trials is due to A. Bradford Hill who designed the first randomized clinical trial evaluating the use of streptomycin in treating tuberculosis in 1946 [9, 12, 13].

Reference [14] provides a good summary of the rationale and justification for the use of randomization in clinical trials. The randomized controlled trial (RCT) has been referred to as “the worst possible design (except for all the rest)” [15], indicating that the benefits of randomization should be evaluated in comparison to what we are left with if we do not randomize. Observational studies suffer from a wide variety of biases that may not be adequately addressed even using state-of-the-art statistical modeling techniques.

The RCT in the medical field has several features that distinguish it from experimental designs in other fields, such as agricultural experiments. In the RCT, the experimental units are humans, and in the medical field often diagnosed with a potentially fatal disease. These subjects are sequentially enrolled for participation in the study at selected study centers, which have relevant expertise for conducting clinical research. Many contemporary clinical trials are run globally, at multiple research institutions. The recruitment period may span several months or even years, depending on a therapeutic indication and the target patient population. Patients who meet study eligibility criteria must sign the informed consent, after which they are enrolled into the study and, for example, randomized to either experimental treatment E or the control treatment C according to the randomization sequence. In this setup, the choice of the randomization design must be made judiciously, to protect the study from experimental biases and ensure validity of clinical trial results.

The first virtue of randomization is that, in combination with allocation concealment and masking, it helps mitigate selection bias due to an investigator’s potential to selectively enroll patients into the study [16]. A non-randomized, systematic design such as a sequence of alternating treatment assignments has a major fallacy: an investigator, knowing an upcoming treatment assignment in a sequence, may enroll a patient who, in their opinion, would be best suited for this treatment. Consequently, one of the groups may contain a greater number of “sicker” patients and the estimated treatment effect may be biased. Systematic covariate imbalances may increase the probability of false positive findings and undermine the integrity of the trial. While randomization alleviates the fallacy of a systematic design, it does not fully eliminate the possibility of selection bias (unless we consider complete randomization for which each treatment assignment is determined by a flip of a coin, which is rarely, if ever used in practice [17]). Commonly, RCTs employ restricted randomization procedures which sequentially balance treatment assignments while maintaining allocation randomness. A popular choice is the permuted block design that controls imbalance by making treatment assignments at random in blocks. To minimize potential for selection bias, one should avoid overly restrictive randomization schemes such as permuted block design with small block sizes, as this is very similar to alternating treatment sequence.

The second virtue of randomization is its tendency to promote similarity of treatment groups with respect to important known, but even more importantly, unknown confounders. If treatment assignments are made at random, then by the law of large numbers, the average values of patient characteristics should be approximately equal in the experimental and the control groups, and any observed treatment difference should be attributed to the treatment effects, not the effects of the study participants [18]. However, one can never rule out the possibility that the observed treatment difference is due to chance, e.g. as a result of random imbalance in some patient characteristics [19]. Despite that random covariate imbalances can occur in clinical trials of any size, such imbalances do not compromise the validity of statistical inference, provided that proper statistical techniques are applied in the data analysis.

Several misconceptions on the role of randomization and balance in clinical trials were documented and discussed by Senn [20]. One common misunderstanding is that balance of prognostic covariates is necessary for valid inference. In fact, different randomization designs induce different extent of balance in the distributions of covariates, and for a given trial there is always a possibility of observing baseline group differences. A legitimate approach is to pre-specify in the protocol the clinically important covariates to be adjusted for in the primary analysis, apply a randomization design (possibly accounting for selected covariates using pre-stratification or some other approach), and perform a pre-planned covariate-adjusted analysis (such as analysis of covariance for a continuous primary outcome), verifying the model assumptions and conducting additional supportive/sensitivity analyses, as appropriate. Importantly, the pre-specified prognostic covariates should always be accounted for in the analysis, regardless whether their baseline differences are present or not [20].

It should be noted that some randomization designs (such as covariate-adaptive randomization procedures) can achieve very tight balance of covariate distributions between treatment groups [21]. While we address randomization within pre-specified stratifications, we do not address more complex covariate- and response-adaptive randomization in this paper.

Finally, randomization plays an important role in statistical analysis of the clinical trial. The most common approach to inference following the RCT is the *invoked population model* [10]. With this approach, one posits that there is an infinite target population of patients with the disease, from which $n$ eligible subjects are sampled in an unbiased manner for the study and are randomized to the treatment groups. Within each group, the responses are assumed to be independent and identically distributed (i.i.d.), and inference on the treatment effect is performed using some standard statistical methodology, e.g. a two sample t-test for normal outcome data. The added value of randomization is that it makes the assumption of i.i.d. errors more feasible compared to a non-randomized study because it introduces a real element of chance in the allocation of patients.

An alternative approach is the *randomization model*, in which the implemented randomization itself forms the basis for statistical inference [10]. Under the null hypothesis of the equality of treatment effects, individual outcomes (which are regarded as not influenced by random variation, i.e. are considered as fixed) are not affected by treatment. Treatment assignments are permuted in all possible ways consistent with the randomization procedure actually used in the trial. The randomization-based *p-*value is the sum of null probabilities of the treatment assignment permutations in the reference set that yield the test statistic values greater than or equal to the experimental value. A randomization-based test can be a useful supportive analysis, free of assumptions of parametric tests and protective against spurious significant results that may be caused by temporal trends [14, 22].

It is important to note that *Bayesian* inference has also become a common statistical analysis in RCTs [23]. Although the inferential framework relies upon subjective probabilities, a study analyzed through a Bayesian framework still relies upon randomization for the other aforementioned virtues [24]. Hence, the randomization considerations discussed herein have broad application.

### What types of randomization methodologies are available?

Randomization is not a single methodology, but a very broad class of design techniques for the RCT [10]. In this paper, we consider only randomization designs for sequential enrollment clinical trials with equal (1:1) allocation in which randomization is not adapted for covariates and/or responses. The simplest procedure for an RCT is complete randomization design (CRD) for which each subject’s treatment is determined by a flip of a fair coin [25]. CRD provides no potential for selection bias (e.g. based on prediction of future assignments) but it can result, with non-negligible probability, in deviations from the 1:1 allocation ratio and covariate imbalances, especially in small samples. This may lead to loss of statistical efficiency (decrease in power) compared to the balanced design. In practice, some restrictions on randomization are made to achieve balanced allocation. Such randomization designs are referred to as *restricted randomization* procedures [26, 27].

Suppose we plan to randomize an even number of subjects $n$ sequentially between treatments E and C. Two basic designs that equalize the final treatment numbers are the random allocation rule (Rand) and the truncated binomial design (TBD), which were discussed in the 1957 paper by Blackwell and Hodges [28]. For Rand, any sequence of exactly $n/2$ E’s and $n/2$ C’s is equally likely. For TBD, treatment assignments are made with probability 0.5 until one of the treatments receives its quota of $n/2$ subjects; thereafter all remaining assignments are made deterministically to the opposite treatment.

A common feature of both Rand and TBD is that they aim at the final balance, whereas at intermediate steps it is still possible to have substantial imbalances, especially if $n$ is large. A long run of a single treatment in a sequence may be problematic if there is a time drift in some important covariate, which can lead to chronological bias [29]. To mitigate this risk, one can further restrict randomization so that treatment assignments are balanced over time. One common approach is the permuted block design (PBD) [30], for which random treatment assignments are made in blocks of size $2b$ ($b$ is some small positive integer), with exactly $b$ allocations to each of the treatments E and C. The PBD is perhaps the oldest (it can be traced back to A. Bradford Hill’s 1951 paper [12]) and the most widely used randomization method in clinical trials. Often its choice in practice is justified by simplicity of implementation and the fact that it is referenced in the authoritative ICH E9 guideline on statistical principles for clinical trials [31]. One major challenge with PBD is the choice of the block size. If $b=1$, then every pair of allocations is balanced, but every even allocation is deterministic. Larger block sizes increase allocation randomness. The use of variable block sizes has been suggested [31]; however, PBDs with variable block sizes are also quite predictable [32]. Another problematic feature of the PBD is that it forces periodic return to perfect balance, which may be unnecessary from the statistical efficiency perspective and may increase the risk of prediction of upcoming allocations.

More recent and better alternatives to the PBD are the *maximum tolerated imbalance* (MTI) procedures [33–41]. These procedures provide stronger encryption of the randomization sequence (i.e. make it more difficult to predict future treatment allocations in the sequence even knowing the current sizes of the treatment groups) while controlling treatment imbalance at a pre-defined threshold throughout the experiment. A general MTI procedure specifies a certain boundary for treatment imbalance, say $b>0$, that cannot be exceeded. If, at a given allocation step the absolute value of imbalance is equal to $b$, then one next allocation is deterministically forced toward balance. This is in contrast to PBD which, after reaching the target quota of allocations for either treatment within a block, forces all subsequent allocations to achieve perfect balance at the end of the block. Some notable MTI procedures are the big stick design (BSD) proposed by Soares and Wu in 1983 [37], the maximal procedure proposed by Berger, Ivanova and Knoll in 2003 [35], the block urn design (BUD) proposed by Zhao and Weng in 2011 [40], just to name a few. These designs control treatment imbalance within pre-specified limits and are more immune to selection bias than the PBD [42, 43].

Another important class of restricted randomization procedures is biased coin designs (BCDs). Starting with the seminal 1971 paper of Efron [44], BCDs have been a hot research topic in biostatistics for 50 years. Efron’s BCD is very simple: at any allocation step, if treatment numbers are balanced, the next assignment is made with probability 0.5; otherwise, the underrepresented treatment is assigned with probability $p$, where $0.5<p\le 1$ is a fixed and pre-specified parameter that determines the tradeoff between balance and randomness. Note that $p=1$ corresponds to PBD with block size 2. If we set $p<1$ (e.g. $p=2/3$), then the procedure has no deterministic assignments and treatment allocation will be concentrated around 1:1 with high probability [44]. Several extensions of Efron’s BCD providing better tradeoff between treatment balance and allocation randomness have been proposed [45–49]; for example, a class of adjustable biased coin designs introduced by Baldi Antognini and Giovagnoli in 2004 [49] unifies many BCDs in a single framework. A comprehensive simulation study comparing different BCDs has been published by Atkinson in 2014 [50].

Finally, urn models provide a useful mechanism for RCT designs [51]. Urn models apply some probabilistic rules to sequentially add/remove balls (representing different treatments) in the urn, to balance treatment assignments while maintaining the randomized nature of the experiment [39, 40, 52–55]. A randomized urn design for balancing treatment assignments was proposed by Wei in 1977 [52]. More novel urn designs, such as the drop-the-loser urn design developed by Ivanova in 2003 [55] have reduced variability and can attain the target treatment allocation more efficiently. Many urn designs involve parameters that can be fine-tuned to obtain randomization procedures with desirable balance/randomness tradeoff [56].

### What are the attributes of a good randomization procedure?

A “good” randomization procedure is one that helps successfully achieve the study objective(s). Kalish and Begg [57] state that the major objective of a comparative clinical trial is to provide a precise and valid comparison. To achieve this, the trial design should be such that it: 1) prevents bias; 2) ensures an efficient treatment comparison; and 3) is simple to implement to minimize operational errors. Table 1 elaborates on these considerations, focusing on restricted randomization procedures for 1:1 randomized trials.

**Table 1:** Considerations for the choice of a restricted randomization procedure

Objective	Desired feature(s) of a randomization procedure
Mitigate potential for selection bias	A procedure should have high degree of randomness.
Mitigate potential for chronological bias.	A procedure should balance treatment assignments over time.
Valid and efficient treatment comparison	A procedure should have established statistical properties, provide strong control of false positive rate and yield unbiased, low variance estimates of the treatment difference. A procedure should preserve the unconditional allocation ratio (e.g. 1:1) at every allocation step and achieve approximately or exactly the target sample sizes per group.
Ease of implementation	Validated statistical software for implementing a randomization procedure must be in place.

Before delving into a detailed discussion, let us introduce some important definitions. Following [10], a *randomization sequence* is a random vector ${{\varvec{\updelta}}}_{n}=({\delta }_{1},\dots ,{\delta }_{n})$, where ${\delta }_{i}=1$, if the *i*th subject is assigned to treatment E or ${\delta }_{i}=0$, if the $i$th subject is assigned to treatment C. A *restricted randomization procedure* can be defined by specifying a probabilistic rule for the treatment assignment of the (*i*+1)st subject, ${\delta }_{i+1}$, given the past allocations ${{\varvec{\updelta}}}_{i}$ for $i\ge 1$. Let ${N}_{E}\left(i\right)={\sum }_{j=1}^{i}{\delta }_{j}$ and ${N}_{C}\left(i\right)=i-{N}_{E}\left(i\right)$ denote the numbers of subjects assigned to treatments E and C, respectively, after $i$ allocation steps. Then $D\left(i\right)={N}_{E}\left(i\right)-{N}_{C}(i)$ is *treatment imbalance* after $i$ allocations. For any $i\ge 1$, $D\left(i\right)$ is a random variable whose probability distribution is determined by the chosen randomization procedure.

#### Balance and randomness

Treatment balance and allocation randomness are two competing requirements in the design of an RCT. Restricted randomization procedures that provide a good tradeoff between these two criteria are desirable in practice.

Consider a trial with sample size $n$. The absolute value of imbalance, $\left|D(i)\right|$ $(i=1,\dots,n)$, provides a measure of deviation from equal allocation after $i$ allocation steps. $\left|D(i)\right|=0$ indicates that the trial is perfectly balanced. One can also consider $\Pr(\vert D\left(i\right)\vert=0)$, the probability of achieving exact balance after $i$ allocation steps. In particular $\Pr(\vert D\left(n\right)\vert=0)$ is the probability that the final treatment numbers are balanced. Two other useful summary measures are the expected imbalance at the $i\mathrm{th}$ step, $E\left|D(i)\right|$ and the expected value of the maximum imbalance of the entire randomization sequence, $E\left(\underset{1\le i\le n}{\mathrm{max}}\left|D\left(i\right)\right|\right)$.

Greater forcing of balance implies lack of randomness. A procedure that lacks randomness may be susceptible to selection bias [16], which is a prominent issue in open-label trials with a single center or with randomization stratified by center, where the investigator knows the sequence of all previous treatment assignments. A classic approach to quantify the degree of susceptibility of a procedure to selection bias is the Blackwell-Hodges model [28]. Let ${G}_{i}=1$ (or 0), if at the $i\mathrm{th}$ allocation step an investigator makes a correct (or incorrect) guess on treatment assignment ${\delta }_{i}$, given past allocations ${{\varvec{\updelta}}}_{i-1}$. Then the predictability of the design at the $i\mathrm{th}$ step is the expected value of ${G}_{i}$, i.e. $E\left(G_i\right)=\Pr(G_i=1)$. Blackwell and Hodges [28] considered the *expected bias factor*, the difference between expected total number of correct guesses of a given sequence of random assignments and the similar quantity obtained from CRD for which treatment assignments are made independently with equal probability: $E(F)=E\left({\sum }_{i=1}^{n}{G}_{i}\right)-n/2$. This quantity is zero for CRD, and it is positive for restricted randomization procedures (greater values indicate higher expected bias). Matts and Lachin [30] suggested taking *expected proportion of deterministic assignments* in a sequence as another measure of lack of randomness.

In the literature, various restricted randomization procedures have been compared in terms of balance and randomness [50, 58, 59]. For instance, Zhao et al. [58] performed a comprehensive simulation study of 14 restricted randomization procedures with different choices of design parameters, for sample sizes in the range of 10 to 300. The key criteria were the maximum absolute imbalance and the correct guess probability. The authors found that the performance of the designs was within a closed region with the boundaries shaped by Efron’s BCD [44] and the big stick design [37], signifying that the latter procedure with a suitably chosen MTI boundary can be superior to other restricted randomization procedures in terms of balance/randomness tradeoff. Similar findings confirming the utility of the big stick design were recently reported by Hilgers et al. [60].

#### Validity and efficiency

*Validity* of a statistical procedure essentially means that the procedure provides correct statistical inference following an RCT. In particular, a chosen statistical test is valid, if it controls the chance of a false positive finding, that is, the pre-specified probability of a type I error of the test is achieved but not exceeded. The strong control of type I error rate is a major prerequisite for any confirmatory RCT. *Efficiency* means high statistical power for detecting meaningful treatment differences (when they exist), and high accuracy of estimation of treatment effects.

Both validity and efficiency are major requirements of any RCT, and both of these aspects are intertwined with treatment balance and allocation randomness. Restricted randomization designs, when properly implemented, provide solid ground for valid and efficient statistical inference. However, a careful consideration of different options can help an investigator to optimize the choice of a randomization procedure for their clinical trial.

Let us start with statistical efficiency. Equal (1:1) allocation frequently maximizes power and estimation precision. To illustrate this, suppose the primary outcomes in the two groups are normally distributed with respective means ${\mu }_{E}$ and ${\mu }_{C}$ and common standard deviation $\sigma >0$. Then the variance of an efficient estimator of the treatment difference ${\mu }_{E}-{\mu }_{C}$ is equal to $V=\frac{4{\sigma }^{2}}{n-{L}_{n}}$, where ${L}_{n}=\frac{{\left|D(n)\right|}^{2}}{n}$ is referred to as *loss* [61]. Clearly, $V$ is minimized when ${L}_{n}=0$, or equivalently, $D\left(n\right)=0$, i.e. the balanced trial.

When the primary outcome follows a more complex statistical model, optimal allocation may be unequal across the treatment groups; however, 1:1 allocation is still nearly optimal for binary outcomes [62, 63], survival outcomes [64], and possibly more complex data types [65, 66]. Therefore, a randomization design that balances treatment numbers frequently promotes efficiency of the treatment comparison.

As regards inferential validity, it is important to distinguish two approaches to statistical inference after the RCT – an *invoked population* model and a *randomization* model [10]. For a given randomization procedure, these two approaches generally produce similar results when the assumption of normal random sampling (and some other assumptions) are satisfied, but the randomization model may be more robust when model assumptions are violated; e.g. when outcomes are affected by a linear time trend [67, 68]. Another important issue that may interfere with validity is selection bias. Some authors showed theoretically that PBDs with small block sizes may result in serious inflation of the type I error rate under a selection bias model [69–71]. To mitigate risk of selection bias, one should ideally take preventative measures, such as blinding/masking, allocation concealment, and avoidance of highly restrictive randomization designs. However, for already completed studies with evidence of selection bias [72], special statistical adjustments are warranted to ensure validity of the results [73–75].

### Implementation aspects

With the current state of information technology, implementation of randomization in RCTs should be straightforward. Validated randomization systems are emerging, and they can handle randomization designs of increasing complexity for clinical trials that are run globally. However, some important points merit consideration.

The first point has to do with how a randomization sequence is generated and implemented. One should distinguish between *advance* and *adaptive* randomization [16]. Here, by “adaptive” randomization we mean “in-real-time” randomization, i.e. when a randomization sequence is generated not upfront, but rather sequentially, as eligible subjects enroll into the study. Restricted randomization procedures are “allocation-adaptive”, in the sense that the treatment assignment of an individual subject is adapted to the history of previous treatment assignments. While in practice the majority of trials with restricted and stratified randomization use randomization schedules pre-generated in advance, there are some circumstances under which “in-real-time” randomization schemes may be preferred; for instance, clinical trials with high cost of goods and/or shortage of drug supply [76].

The advance randomization approach includes the following steps: 1) for the chosen randomization design and sample size $n$, specify the probability distribution on the reference set by enumerating all feasible randomization sequences of length $n$ and their corresponding probabilities; 2) select a sequence at random from the reference set according to the probability distribution; and 3) implement this sequence in the trial. While enumeration of all possible sequences and their probabilities is feasible and may be useful for trials with small sample sizes, the task becomes computationally prohibitive (and unnecessary) for moderate or large samples. In practice, Monte Carlo simulation can be used to approximate the probability distribution of the reference set of all randomization sequences for a chosen randomization procedure.

A limitation of advance randomization is that a sequence of treatment assignments must be generated upfront, and proper security measures (e.g. blinding/masking) must be in place to protect confidentiality of the sequence. With the adaptive or “in-real-time” randomization, a sequence of treatment assignments is generated dynamically as the trial progresses. For many restricted randomization procedures, the randomization rule can be expressed as $\Pr(\delta_{i+1}=1)=\left|F\left\{D\left(i\right)\right\}\right|$, where $F\left\{\cdot \right\}$ is some non-increasing function of $D\left(i\right)$ for any $i\ge 1$. This is referred to as the *Markov property* [77], which makes a procedure easy to implement sequentially. Some restricted randomization procedures, e.g. the maximal procedure [35], do not have the Markov property.

The second point has to do with how the final data analysis is performed. With an invoked population model, the analysis is conditional on the design and the randomization is ignored in the analysis. With a randomization model, the randomization itself forms the basis for statistical inference. Reference [14] provides a contemporaneous overview of randomization-based inference in clinical trials. Several other papers provide important technical details on randomization-based tests, including justification for control of type I error rate with these tests [22, 78, 79]. In practice, Monte Carlo simulation can be used to estimate randomization-based *p-*values [10].

### A roadmap for comparison of restricted randomization procedures

The design of any RCT starts with formulation of the trial objectives and research questions of interest [3, 31]. The choice of a randomization procedure is an integral part of the study design. A structured approach for selecting an appropriate randomization procedure for an RCT was proposed by Hilgers et al. [60]. Here we outline the thinking process one may follow when evaluating different candidate randomization procedures. Our presented roadmap is by no means exhaustive; its main purpose is to illustrate the logic behind some important considerations for finding an “optimal” randomization design for the given trial parameters.

Throughout, we shall assume that the study is designed as a randomized, two-arm comparative trial with 1:1 allocation, with a fixed sample size $n$ that is pre-determined based on budgetary and statistical considerations to obtain a definitive assessment of the treatment effect via the pre-defined hypothesis testing. We start with some general considerations which determine the study design:

- *Sample size (*$n$*).* For small or moderate studies, exact attainment of the target numbers per group may be essential, because even slight imbalance may decrease study power. Therefore, a randomization design in such studies should equalize well the final treatment numbers. For large trials, the risk of major imbalances is less of a concern, and more random procedures may be acceptable.
- *The length of the recruitment period and the trial duration.* Many studies are short-term and enroll participants fast, whereas some other studies are long-term and may have slow patient accrual. In the latter case, there may be time drifts in patient characteristics, and it is important that the randomization design balances treatment assignments over time.
- *Level of blinding (masking): double-blind, single-blind, or open-label.* In double-blind studies with properly implemented allocation concealment the risk of selection bias is low. By contrast, in open-label studies the risk of selection bias may be high, and the randomization design should provide strong encryption of the randomization sequence to minimize prediction of future allocations.
- *Number of study centers.* Many modern RCTs are implemented globally at multiple research institutions, whereas some studies are conducted at a single institution. In the former case, the randomization is often stratified by center and/or clinically important covariates. In the latter case, especially in single-institution open-label studies, the randomization design should be chosen very carefully, to mitigate the risk of selection bias.

An important point to consider is calibration of the design parameters. Many restricted randomization procedures involve parameters, such as the block size in the PBD, the coin bias probability in Efron’s BCD, the MTI threshold, etc. By fine-tuning these parameters, one can obtain designs with desirable statistical properties. For instance, references [80, 81] provide guidance on how to justify the block size in the PBD to mitigate the risk of selection bias or chronological bias. Reference [82] provides a formal approach to determine the “optimal” value of the parameter $p$ in Efron’s BCD in both finite and large samples. The calibration of design parameters can be done using Monte Carlo simulations for the given trial setting.

Another important consideration is the scope of randomization procedures to be evaluated. As we mentioned already, even one method may represent a broad class of randomization procedures that can provide different levels of balance/randomness tradeoff; e.g. Efron’s BCD covers a wide spectrum of designs, from PBD(2) (if $p=1$) to CRD (if $p=0.5$). One may either prefer to focus on finding the “optimal” parameter value for the chosen design, or be more general and include various designs (e.g. MTI procedures, BCDs, urn designs, etc.) in the comparison. This should be done judiciously, on a case-by-case basis, focusing only on the most reasonable procedures. References [50, 58, 60] provide good examples of simulation studies to facilitate comparisons among various restricted randomization procedures for a 1:1 RCT.

In parallel with the decision on the scope of randomization procedures to be assessed, one should decide upon the performance criteria against which these designs will be compared. Among others, one might think about the two competing considerations: treatment balance and allocation randomness. For a trial of size $n$, at each allocation step $i=1,\dots ,n$ one can calculate expected absolute imbalance $E\left|D(i)\right|$ and the probability of correct guess $\Pr(G_i=1)$ as measures of lack of balance and lack of randomness, respectively. These measures can be either calculated analytically (when formulae are available) or through Monte Carlo simulations. Sometimes it may be useful to look at cumulative measures up to the $i\mathrm{th}$ allocation step ($i=1,\dots ,n$); e.g. $\frac{1}{i}{\sum }_{j=1}^{i}E\left|D(j)\right|$ and $\frac1i\sum\nolimits_{j=1}^i\Pr(G_j=1)$. For instance, $\frac{1}{n}{\sum }_{j=1}^{n}{\mathrm{Pr}}({G}_{j}=1)$ is the average correct guess probability for a design with sample size $n$. It is also helpful to visualize the selected criteria. Visualizations can be done in a number of ways; e.g. plots of a criterion vs. allocation step, admissibility plots of two chosen criteria [50, 59], etc. Such visualizations can help evaluate design characteristics, both overall and at intermediate allocation steps. They may also provide insights into the behavior of a particular design for different values of the tuning parameter, and/or facilitate a comparison among different types of designs.

Another way to compare the merits of different randomization procedures is to study their inferential characteristics such as type I error rate and power under different experimental conditions. Sometimes this can be done analytically, but a more practical approach is to use Monte Carlo simulation. The choice of the modeling and analysis strategy will be context-specific. Here we outline some considerations that may be useful for this purpose:

- *Data generating mechanism*. To simulate individual outcome data, some plausible statistical model must be posited. The form of the model will depend on the type of outcomes (e.g. continuous, binary, time-to-event, etc.), covariates (if applicable), the distribution of the measurement error terms, and possibly some additional terms representing selection and/or chronological biases [60].
- *True treatment effects*. At least two scenarios should be considered: under the null hypothesis (${H}_{0}$: treatment effects are the same) to evaluate the type I error rate, and under an alternative hypothesis (${H}_{1}$: there is some true clinically meaningful difference between the treatments) to evaluate statistical power.
- *Randomization designs to be compared*. The choice of candidate randomization designs and their parameters must be made judiciously.
- *Data analytic strategy*. For any study design, one should pre-specify the data analysis strategy to address the primary research question. Statistical tests of significance to compare treatment effects may be parametric or nonparametric, with or without adjustment for covariates.
- *The approach to statistical inference: population model-based or randomization-based*. These two approaches are expected to yield similar results when the population model assumptions are met, but they may be different if some assumptions are violated. Randomization-based tests following restricted randomization procedures will control the type I error at the chosen level if the distribution of the test statistic under the null hypothesis is fully specified by the randomization procedure that was used for patient allocation. This is always the case unless there is a major flaw in the design (such as selection bias whereby the outcome of any individual participant is dependent on treatment assignments of the previous participants).

Overall, there should be a well-thought plan capturing the key questions to be answered, the strategy to address them, the choice of statistical software for simulation and visualization of the results, and other relevant details.

## Results

In this section we present four examples that illustrate how one may approach evaluation of different randomization design options at the study planning stage. Example 1 is based on a hypothetical 1:1 RCT with $n=50$ and a continuous primary outcome, whereas Examples 2, 3, and 4 are based on some real RCTs.

### Example 1: Which restricted randomization procedures are robust and efficient?

Our first example is a hypothetical RCT in which the primary outcome is assumed to be normally distributed with mean ${\mu }_{E}$ for treatment E, mean ${\mu }_{C}$ for treatment C, and common variance ${\sigma }^{2}$. A total of $n$ subjects are to be randomized equally between E and C, and a two-sample t-test is planned for data analysis. Let $\Delta ={\mu }_{E}-{\mu }_{C}$ denote the true mean treatment difference. We are interested in testing a hypothesis ${H}_{0}:\Delta =0$ (treatment effects are the same) vs. ${H}_{1}:\Delta \ne 0$.

The total sample size $n$ to achieve given power at some clinically meaningful treatment difference ${\Delta }_{c}$ while maintaining the chance of a false positive result at level $\alpha$ can be obtained using standard statistical methods [83]. For instance, if ${\Delta }_{c}/\sigma =0.95$, then a design with $n=50$ subjects (25 per arm) provides approximately 91% power of a two-sample t-test to detect a statistically significant treatment difference using 2-sided $\alpha =$ 5%. We shall consider 12 randomization procedures to sequentially randomize $n=50$ subjects in a 1:1 ratio.

1. Random allocation rule – Rand.
2. Truncated binomial design – TBD.
3. Permuted block design with block size of 2 – PBD(2).
4. Permuted block design with block size of 4 – PBD(4).
5. Big stick design [37] with MTI = 3 – BSD(3).
6. Biased coin design with imbalance tolerance [38] with *p *= 2/3 and MTI = 3 – BCDWIT(2/3, 3).
7. Efron’s biased coin design [44] with *p *= 2/3 – BCD(2/3).
8. Adjustable biased coin design [49] with a = 2 – ABCD(2).
9. Generalized biased coin design (GBCD) with $\gamma =1$ [45] – GBCD(1).
10. GBCD with $\gamma =2$ [46] – GBCD(2).
11. GBCD with $\gamma =5$ [47] – GBCD(5).
12. Complete randomization design – CRD.

These 12 procedures can be grouped into five major types. I) Procedures 1, 2, 3, and 4 achieve exact final balance for a chosen sample size (provided the total sample size is a multiple of the block size). II) Procedures 5 and 6 ensure that at any allocation step the absolute value of imbalance is capped at MTI = 3. III) Procedures 7 and 8 are biased coin designs that sequentially adjust randomization according to imbalance measured as the difference in treatment numbers. IV) Procedures 9, 10, and 11 (GBCD’s with $\gamma =$ 1, 2, and 5) are adaptive biased coin designs, for which randomization probability is modified according to imbalance measured as the difference in treatment allocation proportions (larger $\gamma$ implies greater forcing of balance). V) Procedure 12 (CRD) is the most random procedure that achieves balance for large samples.

#### Balance/randomness tradeoff

We first compare the procedures with respect to treatment balance and allocation randomness. To quantify imbalance after $i$ allocations, we consider two measures: expected value of absolute imbalance $E\left|D(i)\right|$, and expected value of loss $E({L}_{i})=E{\left|D(i)\right|}^{2}/i$ [50, 61]. Importantly, for procedures 1, 2, and 3 the final imbalance is always zero, thus $E\left|D(n)\right|\equiv 0$ and $E({L}_{n})\equiv 0$, but at intermediate steps one may have $E\left|D(i)\right|>0$ and $E\left({L}_{i}\right)>0$, for $1\le i<n$. For procedures 5 and 6 with MTI = 3, $E\left({L}_{i}\right)\le 9/i$. For procedures 7 and 8, $E\left({L}_{n}\right)$ tends to zero as $n\to \infty$ [49]. For procedures 9, 10, 11, and 12, as $n\to \infty$, $E\left({L}_{n}\right)$ tends to the positive constants 1/3, 1/5, 1/11, and 1, respectively [47]. We take the cumulative average loss after $n$ allocations as an aggregate measure of imbalance: $Imb\left(n\right)=\frac{1}{n}{\sum }_{i=1}^{n}E\left({L}_{i}\right)$, which takes values in the 0–1 range.

To measure lack of randomness, we consider two measures: expected proportion of correct guesses up to the $i\mathrm{th}$ step, $PCG\left(i\right)=\frac1i\sum\nolimits_{j=1}^i\Pr(G_j=1)$, $i=1,\dots ,n$, and the *forcing index* [47, 84], $FI(i)=\frac{{\sum }_{j=1}^{i}E\left|{\phi }_{j}-0.5\right|}{i/4}$, where $E\left|{\phi }_{j}-0.5\right|$ is the expected deviation of the conditional probability of treatment E assignment at the $j\mathrm{th}$ allocation step (${\phi }_{j}$) from the unconditional target value of 0.5. Note that $PCG\left(i\right)$ takes values in the range from 0.5 for CRD to 0.75 for PBD(2) assuming $i$ is even, whereas $FI(i)$ takes values in the 0–1 range. At the one extreme, we have CRD for which $FI(i)\equiv 0$ because for CRD ${\phi }_{i}=0.5$ for any $i\ge 1$. At the other extreme, we have PBD(2) for which every odd allocation is made with probability 0.5, and every even allocation is deterministic, i.e. made with probability 0 or 1. For PBD(2), assuming $i$ is even, there are exactly $i/2$ pairs of allocations, and so ${\sum }_{j=1}^{i}E\left|{\phi }_{j}-0.5\right|=0.5\cdot i/2=i/4$, which implies that $FI(i)=1$ for PBD(2). For all other restricted randomization procedures one has $0<FI(i)<1$.

A “good” randomization procedure should have low values of both loss and forcing index. Different randomization procedures can be compared graphically. As a balance/randomness tradeoff metric, one can calculate the quadratic distance to the origin (0,0) for the chosen sample size, e.g. $d(n)=\sqrt{{\left\{Imb(n)\right\}}^{2}+{\left\{FI(n)\right\}}^{2}}$ (in our example $n=50$), and the randomization designs can then be ranked such that designs with lower values of $d(n)$ are preferable.

We ran a simulation study of the 12 randomization procedures for an RCT with $n=50$. Monte Carlo average values of absolute imbalance, loss, $Imb\left(i\right)$, $FI\left(i\right)$, and $d(i)$ were calculated for each intermediate allocation step ($i=1,\dots ,50$), based on 10,000 simulations.

**Fig. 1:**
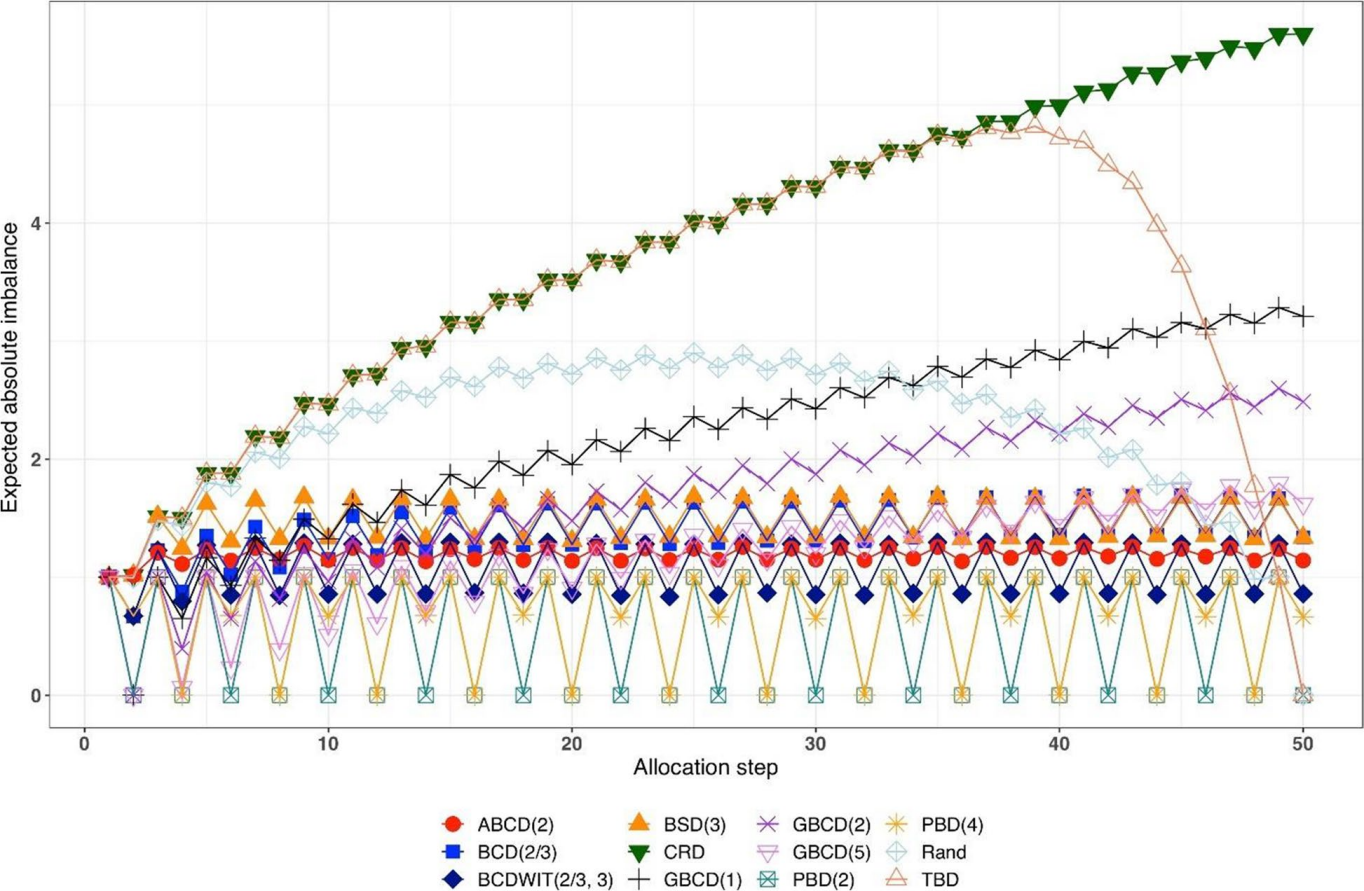
Simulated expected absolute imbalance vs. allocation step for 12 restricted randomization procedures for *n *= 50. Note: PBD(2) and PBD(4) have forced periodicity absolute imbalance of 0, which distinguishes them from MTI procedures

Figure 1 is a plot of expected absolute imbalance vs. allocation step. CRD, GBCD(1), and GBCD(2) show increasing patterns. For TBD and Rand, the final imbalance (when $n=50$) is zero; however, at intermediate steps is can be quite large. For other designs, absolute imbalance is expected to be below 2 at any allocation step up to $n=50$. Note the periodic patterns of PBD(2) and PBD(4); for instance, for PBD(2) imbalance is 0 (or 1) for any even (or odd) allocation.

**Fig. 2:**
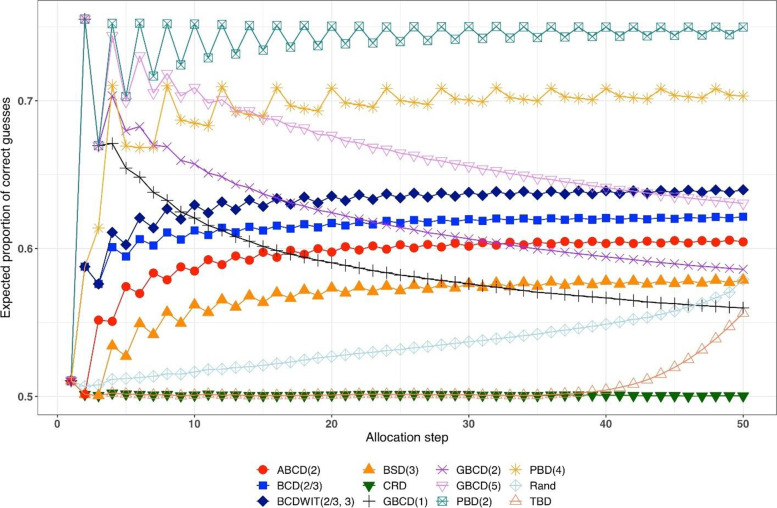
Simulated expected proportion of correct guesses vs. allocation step for 12 restricted randomization procedures for *n *= 50

Figure 2 is a plot of expected proportion of correct guesses vs. allocation step. One can observe that for CRD it is a flat pattern at 0.5; for PBD(2) it fluctuates while reaching the upper limit of 0.75 at even allocation steps; and for ten other designs the values of proportion of correct guesses fall between those of CRD and PBD(2). The TBD has the same behavior up to ~ 40^th^ allocation step, at which the pattern starts increasing. Rand exhibits an increasing pattern with overall fewer correct guesses compared to other randomization procedures. Interestingly, BSD(3) is uniformly better (less predictable) than ABCD(2), BCD(2/3), and BCDWIT(2/3, 3). For the three GBCD procedures, there is a rapid initial increase followed by gradual decrease in the pattern; this makes good sense, because GBCD procedures force greater balance when the trial is small and become more random (and less prone to correct guessing) as the sample size increases.

Table 2 shows the ranking of the 12 designs with respect to the overall performance metric $d(n)=\sqrt{{\left\{Imb(n)\right\}}^{2}+{\left\{FI(n)\right\}}^{2}}$ for $n=50$. BSD(3), GBCD(2) and GBCD(1) are the top three procedures, whereas PBD(2) and CRD are at the bottom of the list.

**Table 2:** Ranking of 12 restricted randomization procedures with respect to balance/randomness tradeoff for a trial with *n *= 50 subjects

Rank	Design	Imb(n)	FI(n)	d(n)
1	BSD(3)	0.226	0.316	0.389
2	GBCD(2)	0.220	0.344	0.409
3	GBCD(1)	0.341	0.240	0.417
4	ABCD(2)	0.170	0.419	0.452
5	GBCD(5)	0.121	0.522	0.536
6	BCD(2/3)	0.233	0.487	0.540
7	BCDWIT(2/3, 3)	0.148	0.560	0.579
8	Rand	0.505	0.318	0.597
9	PBD(4)	0.082	0.813	0.818
10	TBD	0.868	0.225	0.896
11	PBD(2)	0.052	1.000	1.001
12	CRD	1.014	0.000	1.014

**Fig. 3:**
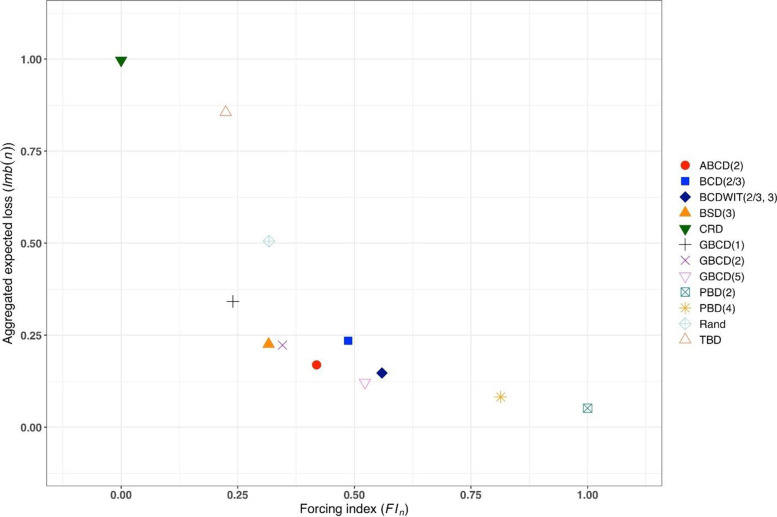
Simulated forcing index (x-axis) vs. aggregate expected loss (y-axis) for 12 restricted randomization procedures for *n *= 50

Figure 3 is a plot of $FI\left(n\right)$ vs. $Imb\left(n\right)$ for $n=50$. One can see the two extremes: CRD that takes the value (0,1), and PBD(2) with the value (1,0). The other ten designs are closer to (0,0).

**Fig. 4:**
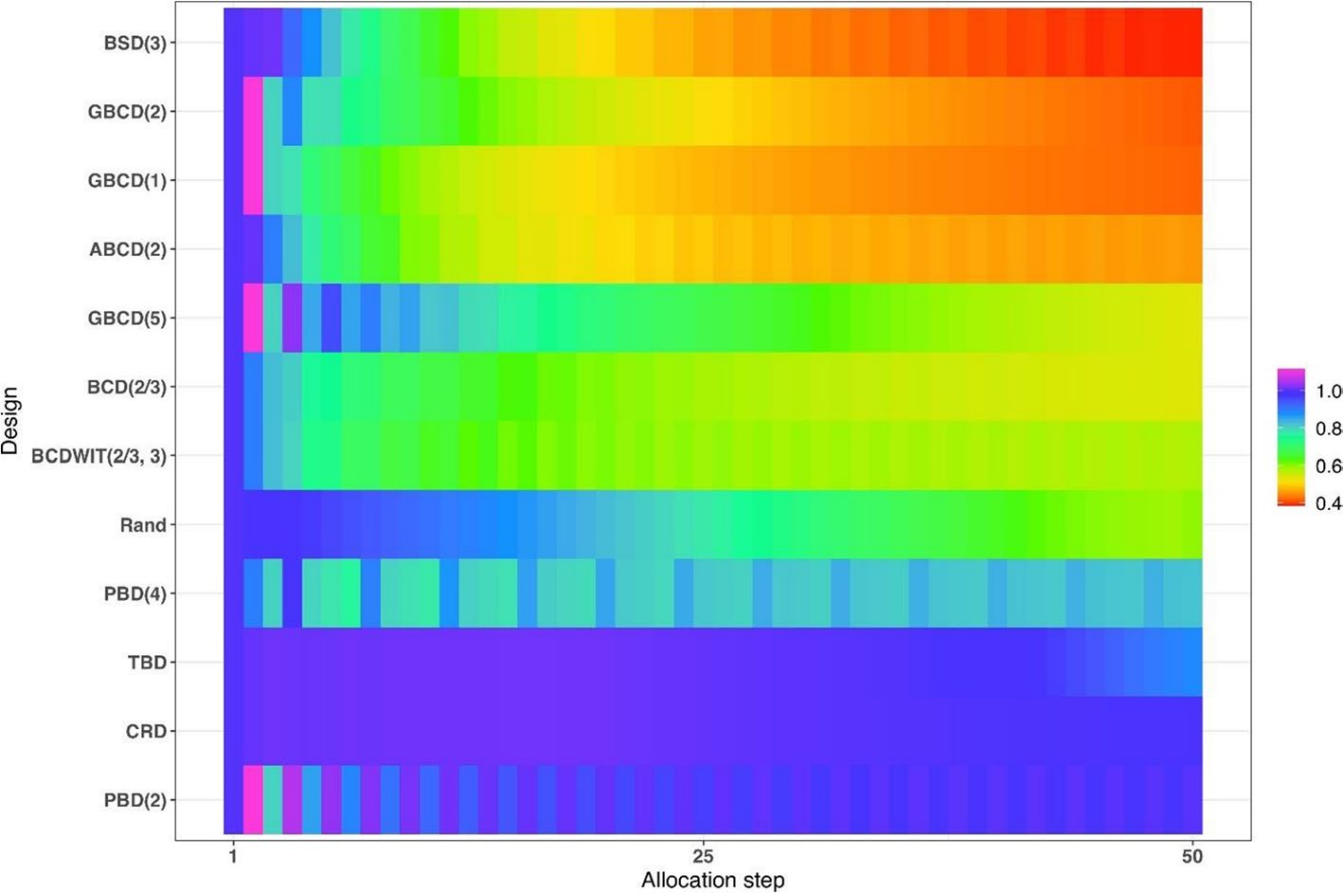
Heatmap of the balance/randomness tradeoff $d\left(i\right)=\sqrt{{\left\{Imb(i)\right\}}^{2}+{\left\{FI(i)\right\}}^{2}}$ vs. allocation step ($i=1,\dots ,50$) for 12 restricted randomization procedures. The procedures are ordered by value of d(50), with smaller values (more red) indicating more optimal performance

Figure 4 is a heat map plot of the metric $d(i)$ for $i=1,\dots ,50$. BSD(3) seems to provide overall best tradeoff between randomness and balance throughout the study.

#### Inferential characteristics: type I error rate and power

Our next goal is to compare the chosen randomization procedures in terms of validity (control of the type I error rate) and efficiency (power). For this purpose, we assumed the following data generating mechanism: for the $i\mathrm{th}$ subject, conditional on the treatment assignment ${\delta }_{i}$, the outcome ${Y}_{i}$ is generated according to the model

1 $${Y}_{i}={\delta }_{i}{\mu }_{E}+\left(1-{\delta }_{i}\right){\mu }_{C}+{u}_{i}+{\varepsilon }_{i}, i=1,\dots ,n$$

where ${u}_{i}$ is an unknown term associated with the $i\mathrm{th}$ subject and ${\varepsilon }_{i}$’s are i.i.d. measurement errors. We shall explore the following four models:

- ***M1: Normal random sampling*: **${u}_{i}\equiv 0$ and ${\varepsilon }_{i}\sim$ i.i.d. N(0,1), $i=1,\dots ,n$. This corresponds to a standard setup for a two-sample t-test under a population model.
- ***M2: Linear trend*: **${u}_{i}=\frac{5i}{n+1}$ and ${\varepsilon }_{i}\sim$ i.i.d. N(0,1), $i=1,\dots ,n$. In this model, the outcomes are affected by a linear trend over time [67].
- ***M3: Cauchy errors*: **${u}_{i}\equiv 0$ and ${\varepsilon }_{i}\sim$ i.i.d. Cauchy(0,1), $i=1,\dots ,n$. In this setup, we have a misspecification of the distribution of measurement errors.
- ***M4: Selection bias*: **${u}_{i+1}=-\nu \cdot sign\left\{D\left(i\right)\right\}$, $i=0,\dots ,n-1$, with the convention that $D\left(0\right)=0$. Here, $\nu >0$ is the “bias effect” (in our simulations we set $\nu =0.5$). We also assume that ${\varepsilon }_{i}\sim$ i.i.d. N(0,1), $i=1,\dots ,n$. In this setup, at each allocation step the investigator attempts to intelligently guess the upcoming treatment assignment and selectively enroll a patient who, in their view, would be most suitable for the upcoming treatment. The investigator uses the “convergence” guessing strategy [28], that is, guess the treatment as one that has been less frequently assigned thus far, or make a random guess in case the current treatment numbers are equal. Assuming that the investigator favors the experimental treatment and is interested in demonstrating its superiority over the control, the biasing mechanism is as follows: at the $(i+1)$ st step, a “healthier” patient is enrolled, if $D\left(i\right)<0$ (${u}_{i+1}=0.5$); a “sicker” patient is enrolled, if $D\left(i\right)>0$ (${u}_{i+1}=-0.5$); or a “regular” patient is enrolled, if $D\left(i\right)=0$ (${u}_{i+1}=0$).

We consider three statistical test procedures:

- ***T1: Two-sample t-test*:** The test statistic is $t=\frac{{\overline{Y} }_{E}-{\overline{Y} }_{C}}{\sqrt{{S}_{p}^{2}\left(\frac{1}{{N}_{E}\left(n\right)}+\frac{1}{{N}_{C}\left(n\right)}\right)}}$, where ${\overline{Y} }_{E}=\frac{1}{{N}_{E}\left(n\right)}{\sum }_{i=1}^{n}{{\delta }_{i}Y}_{i}$ and ${\overline{Y} }_{C}=\frac{1}{{N}_{C}\left(n\right)}{\sum }_{i=1}^{n}{(1-\delta }_{i}){Y}_{i}$ are the treatment sample means, ${N}_{E}\left(n\right)={\sum }_{i=1}^{n}{\delta }_{i}$ and ${N}_{C}\left(n\right)=n-{N}_{E}\left(n\right)$ are the observed group sample sizes, and ${S}_{p}^{2}$ is a pooled estimate of variance, where ${S}_{p}^{2}=\frac{1}{n-2}\left({\sum }_{i=1}^{n}{\delta }_{i}{\left({Y}_{i}-{\overline{Y} }_{E}\right)}^{2}+{\sum }_{i=1}^{n}(1-{\delta }_{i}){\left({Y}_{i}-{\overline{Y} }_{C}\right)}^{2}\right)$. Then ${H}_{0}:\Delta =0$ is rejected at level $\alpha$, if $\left|t\right|>{t}_{1-\frac{\alpha }{2}, n-2}$, the 100($1-\frac{\alpha }{2}$)th percentile of the t-distribution with $n-2$ degrees of freedom.
- ***T2: Randomization-based test using mean difference*:** Let ${{\varvec{\updelta}}}_{obs}$ and ${{\varvec{y}}}_{obs}$ denote, respectively the observed sequence of treatment assignments and responses, obtained from the trial using randomization procedure $\mathfrak{R}$. We first compute the observed mean difference ${S}_{obs}=S\left({{\varvec{\updelta}}}_{obs},{{\varvec{y}}}_{obs}\right)={\overline{Y} }_{E}-{\overline{Y} }_{C}$. Then we use Monte Carlo simulation to generate $L$ randomization sequences of length $n$ using procedure $\mathfrak{R}$, where $L$ is some large number. For the $\ell\mathrm{th}$ generated sequence, ${{\varvec{\updelta}}}_{\ell}$, compute ${S}_{\ell}=S({{\varvec{\updelta}}}_{\ell},{{\varvec{y}}}_{obs})$, where ${\ell}=1,\dots ,L$. The proportion of sequences for which ${S}_{\ell}$ is at least as extreme as ${S}_{obs}$ is computed as $\widehat{P}=\frac{1}{L}{\sum }_{{\ell}=1}^{L}1\left\{\left|{S}_{\ell}\right|\ge \left|{S}_{obs}\right|\right\}$. Statistical significance is declared, if $\widehat{P}<\alpha$.
- ***T3: Randomization-based test based on ranks*:** This test procedure follows the same logic as T2, except that the test statistic is calculated based on ranks. Given the vector of observed responses ${{\varvec{y}}}_{obs}=({y}_{1},\dots ,{y}_{n})$, let ${a}_{jn}$ denote the rank of ${y}_{j}$ among the elements of ${{\varvec{y}}}_{obs}$. Let ${\overline a}_n$ denote the average of ${a}_{jn}$’s, and let ${\boldsymbol a}_n=\left(a_{1n}-{\overline a}_n,...,\alpha_{nn}-{\overline a}_n\right)\boldsymbol'$. Then a linear rank test statistic has the form ${S}_{obs}={{\varvec{\updelta}}}_{obs}^{\boldsymbol{^{\prime}}}{{\varvec{a}}}_{n}={\sum }_{i=1}^{n}{\delta }_{i}({a}_{in}-{\overline{a} }_{n})$.

We consider four scenarios of the true mean difference $\Delta ={\mu }_{E}-{\mu }_{C}$, which correspond to the Null case ($\Delta =0$), and three choices of $\Delta >0$ which correspond to Alternative 1 (power ~ 70%), Alternative 2 (power ~ 80%), and Alternative 3 (power ~ 90%). In all cases, $n=50$ was used.

**Fig. 5:**
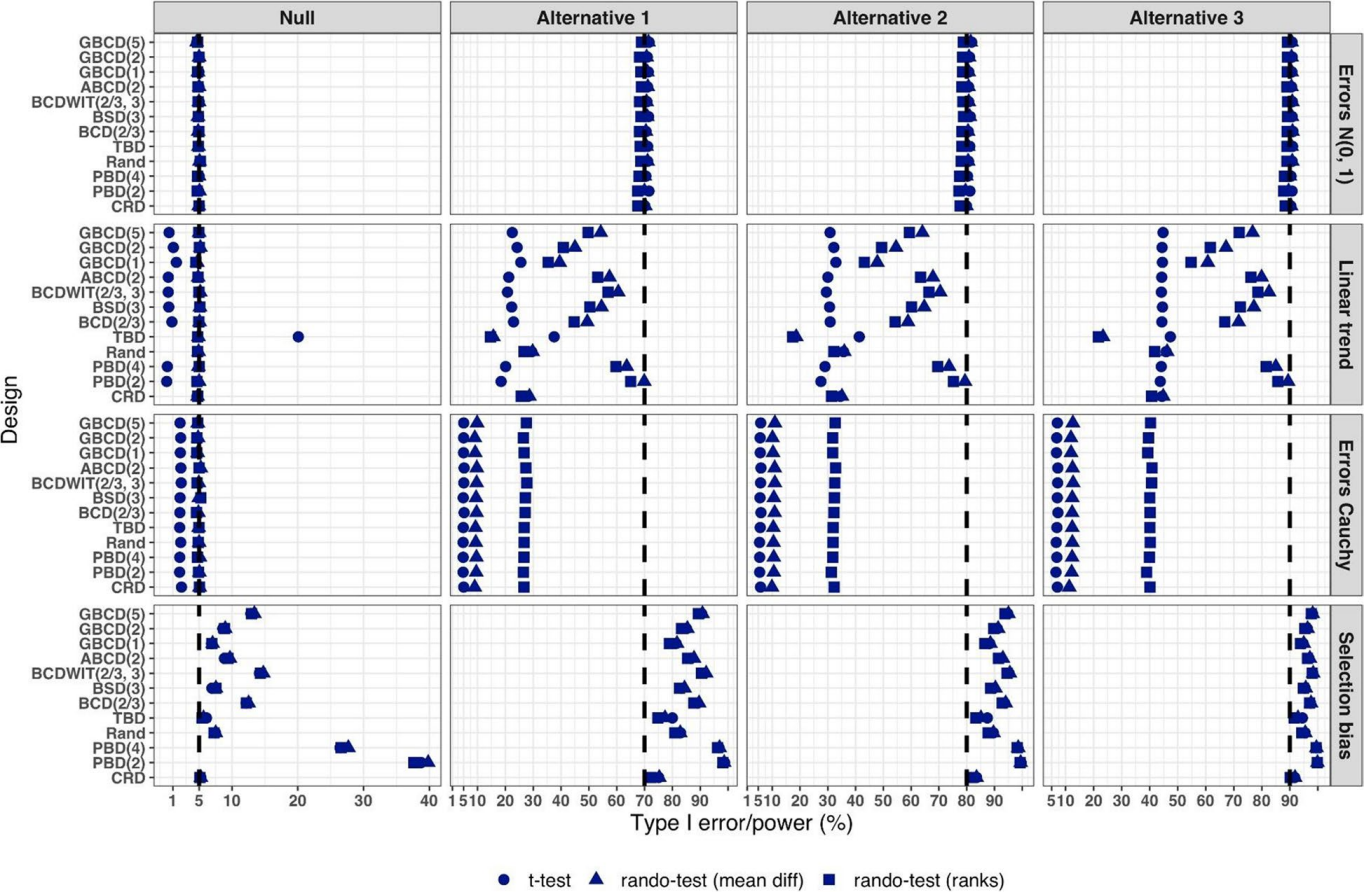
Simulated type I error rate and power of 12 restricted randomization procedures. Four models for the data generating mechanism of the primary outcome (M1: Normal random sampling; M2: Linear trend; M3: Errors Cauchy; and M4: Selection bias). Four scenarios for the treatment mean difference (Null; Alternatives 1, 2, and 3). Three statistical tests (T1: two-sample t-test; T2: randomization-based test using mean difference; T3: randomization-based test using ranks)

Figure 5 summarizes the results of a simulation study comparing 12 randomization designs, under 4 models for the outcome (M1, M2, M3, and M4), 4 scenarios for the mean treatment difference (Null, and Alternatives 1, 2, and 3), using 3 statistical tests (T1, T2, and T3). The operating characteristics of interest are the type I error rate under the Null scenario and the power under the Alternative scenarios. Each scenario was simulated 10,000 times, and each randomization-based test was computed using $L=\mathrm{10,000}$ sequences.

From Fig. 5, under the normal random sampling model (M1), all considered randomization designs have similar performance: they maintain the type I error rate and have similar power, with all tests. In other words, when population model assumptions are satisfied, any combination of design and analysis should work well and yield reliable and consistent results.

Under the “linear trend” model (M2), the designs have differential performance. First of all, under the Null scenario, only Rand and CRD maintain the type I error rate at 5% with all three tests. For TBD, the t-test is anticonservative, with type I error rate ~ 20%, whereas for nine other procedures the t-test is conservative, with type I error rate in the range 0.1–2%. At the same time, for all 12 designs the two randomization-based tests maintain the nominal type I error rate at 5%. These results are consistent with some previous findings in the literature [67, 68]. As regards power, it is reduced significantly compared to the normal random sampling scenario. The t-test seems to be most affected and the randomization-based test using ranks is most robust for a majority of the designs. Remarkably, for CRD the power is similar with all three tests. This signifies the usefulness of randomization-based inference in situations when outcome data are subject to a linear time trend, and the importance of applying randomization-based tests at least as supplemental analyses to likelihood-based test procedures.

Under the “Cauchy errors” model (M3), all designs perform similarly: the randomization-based tests maintain the type I error rate at 5%, whereas the t-test deflates the type I error to 2%. As regards power, all designs also have similar, consistently degraded performance: the t-test is least powerful, and the randomization-based test using ranks has highest power. Overall, under misspecification of the error distribution a randomization-based test using ranks is most appropriate; yet one should acknowledge that its power is still lower than expected.

Under the “selection bias” model (M4), the 12 designs have differential performance. The only procedure that maintained the type I error rate at 5% with all three tests was CRD. For eleven other procedures, inflations of the type I error were observed. In general, the more random the design, the less it was affected by selection bias. For instance, the type I error rate for TBD was ~ 6%; for Rand, BSD(3), and GBCD(1) it was ~ 7.5%; for GBCD(2) and ABCD(2) it was ~ 8–9%; for Efron’s BCD(2/3) it was ~ 12.5%; and the most affected design was PBD(2) for which the type I error rate was ~ 38–40%. These results are consistent with the theory of Blackwell and Hodges [28] which posits that TBD is least susceptible to selection bias within a class of restricted randomization designs that force exact balance. Finally, under M4, statistical power is inflated by several percentage points compared to the normal random sampling scenario without selection bias.

We performed additional simulations to assess the impact of the bias effect $\nu$ under selection bias model. The same 12 randomization designs and three statistical tests were evaluated for a trial with $n=50$ under the Null scenario ($\Delta =0$), for $\nu$ in the range of 0 (no bias) to 1 (strong bias). Figure S1 in the Supplementary Materials shows that for all designs but CRD, the type I error rate is increasing in $\nu$, with all three tests. The magnitude of the type I error inflation is different across the restricted randomization designs; e.g. for TBD it is minimal, whereas for more restrictive designs it may be large, especially for $\nu \ge 0.4$. PBD(2) is particularly vulnerable: for $\nu$ in the range 0.4–1, its type I error rate is in the range 27–90% (for the nominal $\alpha =5$%).

In summary, our Example 1 includes most of the key ingredients of the roadmap for assessment of competing randomization designs which was described in the “Methods” section. For the chosen experimental scenarios, we evaluated CRD and several restricted randomization procedures, some of which belonged to the same class but with different values of the parameter (e.g. GBCD with $\gamma =1, 2, 5$). We assessed two measures of imbalance, two measures of lack of randomness (predictability), and a metric that quantifies balance/randomness tradeoff. Based on these criteria, we found that BSD(3) provides overall best performance. We also evaluated type I error and power of selected randomization procedures under several treatment response models. We have observed important links between balance, randomness, type I error rate and power. It is beneficial to consider all these criteria simultaneously as they may complement each other in characterizing statistical properties of randomization designs. In particular, we found that a design that lacks randomness, such as PBD with blocks of 2 or 4, may be vulnerable to selection bias and lead to inflations of the type I error. Therefore, these designs should be avoided, especially in open-label studies. As regards statistical power, since all designs in this example targeted 1:1 allocation ratio (which is optimal if the outcomes are normally distributed and have between-group constant variance), they had very similar power of statistical tests in most scenarios except for the one with chronological bias. In the latter case, randomization-based tests were more robust and more powerful than the standard two-sample t-test under the population model assumption.

Overall, while Example 1 is based on a hypothetical 1:1 RCT, its true purpose is to showcase the thinking process in the application of our general roadmap. The following three examples are considered in the context of real RCTs.

### Example 2: How can we reduce predictability of a randomization procedure and lower the risk of selection bias?

Selection bias can arise if the investigator can intelligently guess at least part of the randomization sequence yet to be allocated and, on that basis, preferentially and strategically assigns study subjects to treatments. Although it is generally not possible to prove that a particular study has been infected with selection bias, there are examples of published RCTs that do show some evidence to have been affected by it. Suspect trials are, for example, those with strong observed baseline covariate imbalances that consistently favor the active treatment group [16]. In what follows we describe an example of an RCT where the stratified block randomization procedure used was vulnerable to potential selection biases, and discuss potential alternatives that may reduce this vulnerability.

Etanercept was studied in patients aged 4 to 17 years with polyarticular juvenile rheumatoid arthritis [85]. The trial consisted of two parts. During the first, open-label part of the trial, patients received etanercept twice weekly for up to three months. Responders from this initial part of the trial were then randomized, at a 1:1 ratio, in the second, double-blind, placebo-controlled part of the trial to receive etanercept or placebo for four months or until a flare of the disease occurred. The primary efficacy outcome, the proportion of patients with disease flare, was evaluated in the double-blind part. Among the 51 randomized patients, 21 of the 26 placebo patients (81%) withdrew because of disease flare, compared with 7 of the 25 etanercept patients (28%), yielding a *p-*value of 0.003.

Regulatory review by the Food and Drug Administrative (FDA) identified vulnerability to selection biases in the study design of the double-blind part and potential issues in study conduct. These findings were succinctly summarized in [16] (pp.51–52).

Specifically, randomization was stratified by study center and number of active joints (≤ 2 vs. > 2, referred to as “few” or “many” in what follows), with blocked randomization within each stratum using a block size of two. Furthermore, randomization codes in corresponding “few” and “many” blocks within each study center were mirror images of each other. For example, if the first block within the “few” active joints stratum of a given center is “placebo followed by etanercept”, then the first block within the “many” stratum of the same center would be “etanercept followed by placebo”. While this appears to be an attempt to improve treatment balance in this small trial, unblinding of one treatment assignment may lead to deterministic predictability of three upcoming assignments. While the double-blind nature of the trial alleviated this concern to some extent, it should be noted that all patients did receive etanercept previously in the initial open-label part of the trial. Chances of unblinding may not be ignorable if etanercept and placebo have immediately evident different effects or side effects. The randomized withdrawal design was appropriate in this context to improve statistical power in identifying efficacious treatments, but the specific randomization procedure used in the trial increased vulnerability to selection biases if blinding cannot be completely maintained.

FDA review also identified that four patients were randomized from the wrong “few” or “many” strata, in three of which (3/51 = 5.9%) it was foreseeable that the treatment received could have been reversed compared to what the patient would have received if randomized in the correct stratum. There were also some patients randomized out of order. Imbalance in baseline characteristics were observed in age (mean ages of 8.9 years in the etanercept arm vs. that of 12.2 years in the placebo arm) and corticosteroid use at baseline (50% vs. 24%).

While the authors [85] concluded that “The unequal randomization did not affect the study results”, and indeed it was unknown whether the imbalance was a chance occurrence or in part caused by selection biases, the trial could have used better alternative randomization procedures to reduce vulnerability to potential selection bias. To illustrate the latter point, let us compare predictability of two randomization procedures – permuted block design (PBD) and big stick design (BSD) for several values of the maximum tolerated imbalance (MTI). We use BSD here for the illustration purpose because it was found to provide a very good balance/randomness tradeoff based on our simulations in Example 1. In essence, BSD provides the same level of imbalance control as PBD but with stronger encryption.

Table 3 reports two metrics for PBD and BSD: proportion of deterministic assignments within a randomization sequence, and excess correct guess probability. The latter metric is the absolute increase in proportion of correct guesses for a given procedure over CRD that has 50% probability of correct guesses under the “optimal guessing strategy”.^1^ Note that for MTI = 1, BSD is equivalent to PBD with blocks of two. However, by increasing MTI, one can substantially decrease predictability. For instance, going from MTI = 1 in the BSD to an MTI of 2 or 3 (two bottom rows), the proportion of deterministic assignments decreases from 50% to 25% and 16.7%, respectively, and excess correct guess probability decreases from 25% to 12.5% and 8.3%, which is a substantial reduction in risk of selection bias. In addition to simplicity and lower predictability for the same level of MTI control, BSD has another important advantage: investigators are not accustomed to it (as they are to the PBD), and therefore it has potential for complete elimination of prediction through thwarting enough early prediction attempts.

**Table 3:** Predictability of permuted block design (PBD) and big stick design (BSD) for different values of maximum tolerated imbalance (MTI)

MTI	Proportion of Deterministic Assignments		Excess Correct Guess Probability	
	**PBD**	**BSD**	**PBD**	**BSD**
1	50%	50%	25%	25%
2	33.3%	25%	20.8%	12.5%
3	25%	16.7%	18.3%	8.3%

Our observations here are also generalizable to other MTI randomization methods, such as the maximal procedure [35], Chen’s designs [38, 39], block urn design [40], just to name a few. MTI randomization procedures can be also used as building elements for more complex stratified randomization schemes [86].

### Example 3: How can we mitigate risk of chronological bias?

Chronological bias may occur if a trial recruitment period is long, and there is a drift in some covariate over time that is subsequently not accounted for in the analysis [29]. To mitigate risk of chronological bias, treatment assignments should be balanced over time. In this regard, the ICH E9 guideline has the following statement [31]:

“...Although unrestricted randomisation is an acceptable approach, some advantages can generally be gained by randomising subjects in blocks. This helps to increase the comparability of the treatment groups, particularly when subject characteristics may change over time, as a result, for example, of changes in recruitment policy. It also provides a better guarantee that the treatment groups will be of nearly equal size...”

While randomization in blocks of two ensures best balance, it is highly predictable. In practice, a sensible tradeoff between balance and randomness is desirable. In the following example, we illustrate the issue of chronological bias in the context of a real RCT.

Altman and Royston [87] gave several examples of clinical studies with hidden time trends. For instance, an RCT to compare azathioprine versus placebo in patients with primary biliary cirrhosis (PBC) with respect to overall survival was an international, double-blind, randomized trial including 248 patients of whom 127 received azathioprine and 121 placebo [88]. The study had a recruitment period of 7 years. A major prognostic factor for survival was the serum bilirubin level on entry to the trial. Altman and Royston [87] provided a cusum plot of log bilirubin which showed a strong decreasing trend over time – patients who entered the trial later had, on average, lower bilirubin levels, and therefore better prognosis. Despite that the trial was randomized, there was some evidence of baseline imbalance with respect to serum bilirubin between azathioprine and placebo groups. The analysis using Cox regression adjusted for serum bilirubin showed that the treatment effect of azathioprine was statistically significant (*p *= 0.01), with azathioprine reducing the risk of dying to 59% of that observed during the placebo treatment.

**Fig. 6:**
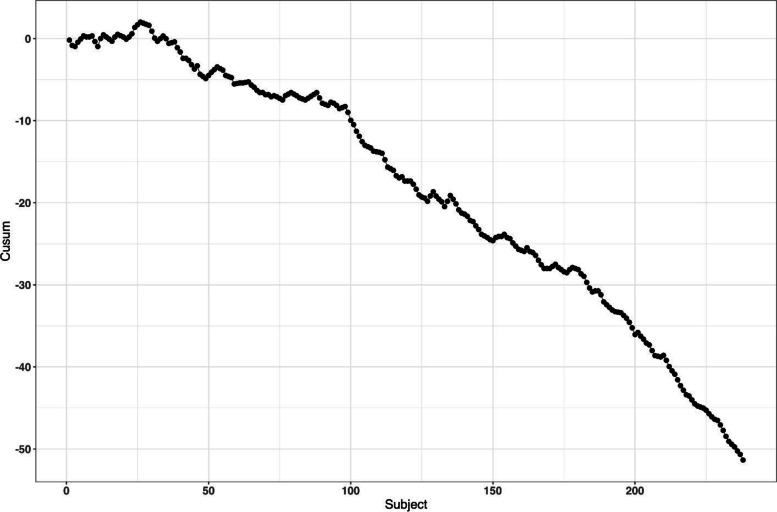
Cusum plot of baseline log serum bilirubin level of 248 subjects from the azathioprine trial, reproduced from Fig. 1 of Altman and Royston [87]

The azathioprine trial [88] provides a very good example for illustrating importance of both the choice of a randomization design and a subsequent statistical analysis. We evaluated several randomization designs and analysis strategies under the given time trend through simulation. Since we did not have access to the patient level data from the azathioprine trial, we simulated a dataset of serum bilirubin values from 248 patients that resembled that in the original paper (Fig. 1 in [87]); see Fig. 6 below.

For the survival outcomes, we use the following data generating mechanism [71, 89]: let ${h}_{i}(t,{\delta }_{i})$ denote the hazard function of the $i\mathrm{th}$ patient at time $t$ such that

2 $${h}_{i}\left(t,{\delta }_{i}\right)={h}_{c}\left(t\right)\mathrm{exp}\left({\delta}_{i}\mathrm{log}\;HR+{u}_{i}\right),\;i=1,\dots ,248$$

where ${h}_{c}(t)$ is an unspecified baseline hazard, $\log HR$ is the true value of the log-transformed hazard ratio, and ${u}_{i}$ is the log serum bilirubin of the $i\mathrm{th}$ patient at study entry.

Our main goal is to evaluate the impact of the time trend in bilirubin on the type I error rate and power. We consider seven randomization designs: CRD, Rand, TBD, PBD(2), PBD(4), BSD(3), and GBCD(2). The latter two designs were found to be the top two performing procedures based on our simulation results in Example 1 (cf. Table 2). PBD(4) is the most commonly used procedure in clinical trial practice. Rand and TBD are two designs that ensure exact balance in the final treatment numbers. CRD is the most random design, and PBD(2) is the most balanced design.

To evaluate both type I error and power, we consider two values for the true treatment effect: $HR=1$ (Null) and $HR=0.6$ (Alternative). For data analysis, we use the Cox regression model, either with or without adjustment for serum bilirubin. Furthermore, we assess two approaches to statistical inference: population model-based and randomization-based. For the sake of simplicity, we let ${h}_{c}\left(t\right)\equiv 1$ (exponential distribution) and assume no censoring when simulating the data.

For each combination of the design, experimental scenario, and data analysis strategy, a trial with 248 patients was simulated 10,000 times. Each randomization-based test was computed using $L=\mathrm{1,000}$ sequences. In each simulation, we used the same time trend in serum bilirubin as described. Through simulation, we estimated the probability of a statistically significant baseline imbalance in serum bilirubin between azathioprine and placebo groups, type I error rate, and power.

First, we observed that the designs differ with respect to their potential to achieve baseline covariate balance under the time trend. For instance, probability of a statistically significant group difference on serum bilirubin (two-sided *P *< 0.05) is ~ 24% for TBD, ~ 10% for CRD, ~ 2% for GBCD(2), ~ 0.9% for Rand, and ~ 0% for BSD(3), PBD(4), and PBD(2).

Second, a failure to adjust for serum bilirubin in the analysis can negatively impact statistical inference. Table 4 shows the type I error and power of statistical analyses unadjusted and adjusted for serum bilirubin, using population model-based and randomization-based approaches.

**Table 4:** Type I error and power of seven randomization designs under a time trend

	Type I error rate		Power	
	**Without adjustment for serum bilirubin**	**With adjustment for serum bilirubin**	**Without adjustment for serum bilirubin**	**With adjustment for serum bilirubin**
Population model-based approach to statistical inference				
CRD	0.0481	0.0504	0.6114	0.9694
Rand	0.0517	0.0511	0.6193	0.9701
TBD	0.1451	0.0511	0.5856	0.9702
PBD(2)	0.0064	0.0511	0.6540	0.9704
PBD(4)	0.0073	0.0518	0.6612	0.9688
BSD(3)	0.0084	0.0541	0.6547	0.9697
GBCD(2)	0.0185	0.0546	0.6367	0.9699
Randomization-based approach to statistical inference				
CRD	0.049	0.052	0.617	0.970
Rand	0.047	0.048	0.602	0.973
TBD	0.047	0.048	0.367	0.968
PBD(2)	0.048	0.048	0.901	0.969
PBD(4)	0.047	0.047	0.874	0.971
BSD(3)	0.048	0.051	0.860	0.964
GBCD(2)	0.050	0.049	0.803	0.971

If we look at the type I error for the population model-based, unadjusted analysis, we can see that only CRD and Rand are valid (maintain the type I error rate at 5%), whereas TBD is anticonservative (~ 15% type I error) and PBD(2), PBD(4), BSD(3), and GBCD(2) are conservative (~ 1–2% type I error). These findings are consistent with the ones for the two-sample t-test described earlier in the current paper, and they agree well with other findings in the literature [67]. By contrast, population model-based covariate-adjusted analysis is valid for all seven randomization designs. Looking at the type I error for the randomization-based analyses, all designs yield consistent valid results (~ 5% type I error), with or without adjustment for serum bilirubin.

As regards statistical power, unadjusted analyses are substantially less powerful then the corresponding covariate-adjusted analysis, for all designs with either population model-based or randomization-based approaches. For the population model-based, unadjusted analysis, the designs have ~ 59–65% power, whereas than the corresponding covariate-adjusted analyses have ~ 97% power. The most striking results are observed with the randomization-based approach: the power of unadjusted analysis is quite different across seven designs: it is ~ 37% for TBD, ~ 60–61% for CRD and Rand, ~ 80–87% for BCD(3), GBCD(2), and PBD(4), and it is ~ 90% for PBD(2). Thus, PBD(2) is the most powerful approach if a time trend is present, statistical analysis strategy is randomization-based, and no adjustment for time trend is made. Furthermore, randomization-based covariate-adjusted analyses have ~ 97% power for all seven designs. Remarkably, the power of covariate-adjusted analysis is identical for population model-based and randomization-based approaches.

Overall, this example highlights the importance of covariate-adjusted analysis, which should be straightforward if a covariate affected by a time trend is known (e.g. serum bilirubin in our example). If a covariate is unknown or hidden, then unadjusted analysis following a conventional test may have reduced power and distorted type I error (although the designs such as CRD and Rand do ensure valid statistical inference). Alternatively, randomization-based tests can be applied. The resulting analysis will be valid but may be potentially less powerful. The degree of loss in power following randomization-based test depends on the randomization design: designs that force greater treatment balance over time will be more powerful. In fact, PBD(2) is shown to be most powerful under such circumstances; however, as we have seen in Example 1 and Example 2, a major deficiency of PBD(2) is its vulnerability to selection bias. From Table 4, and taking into account the earlier findings in this paper, BSD(3) seems to provide a very good risk mitigation strategy against unknown time trends.

### Example 4: How do we design an RCT with a very small sample size?

In our last example, we illustrate the importance of the careful choice of randomization design and subsequent statistical analysis in a nonstandard RCT with small sample size. Due to confidentiality and because this study is still in conduct, we do not disclose all details here except for that the study is an ongoing phase II RCT in a very rare and devastating autoimmune disease in children.

The study includes three periods: an open-label single-arm active treatment for 28 weeks to identify treatment responders (Period 1), a 24-week randomized treatment withdrawal period to primarily assess the efficacy of the active treatment vs. placebo (Period 2), and a 3-year long-term safety, open-label active treatment (Period 3). Because of a challenging indication and the rarity of the disease, the study plans to enroll up to 10 male or female pediatric patients in order to randomize 8 patients (4 per treatment arm) in Period 2 of the study. The primary endpoint for assessing the efficacy of active treatment versus placebo is the proportion of patients with disease flare during the 24-week randomized withdrawal phase. The two groups will be compared using Fisher’s exact test. In case of a successful outcome, evidence of clinical efficacy from this study will be also used as part of a package to support the claim for drug effectiveness.

Very small sample sizes are not uncommon in clinical trials of rare diseases [90, 91]. Naturally, there are several methodological challenges for this type of study. A major challenge is generalizability of the results from the RCT to a population. In this particular indication, no approved treatment exists, and there is uncertainty on disease epidemiology and the exact number of patients with the disease who would benefit from treatment (patient horizon). Another challenge is the choice of the randomization procedure and the primary statistical analysis. In this study, one can enumerate upfront all 25 possible outcomes: {0, 1, 2, 3, 4} responders on active treatment, and {0, 1, 2, 3, 4} responders on placebo, and create a chart quantifying the level of evidence (*p-*value) for each experimental outcome, and the corresponding decision. Before the trial starts, a discussion with the regulatory agency is warranted to agree upon on what level of evidence must be achieved in order to declare the study a “success”.

Let us perform a hypothetical planning for the given study. Suppose we go with a standard population-based approach, for which we test the hypothesis ${H}_{0}:{p}_{E}={p}_{C}$ vs. ${H}_{0}:{p}_{E}>{p}_{C}$ (where ${p}_{E}$ and ${p}_{C}$ stand for the true success rates for the experimental and control group, respectively) using Fisher’s exact test. Table 5 provides 1-sided *p-*values of all possible experimental outcomes. One could argue that a *p-*value < 0.1 may be viewed as a convincing level of evidence for this study. There are only 3 possibilities that can lead to this outcome: 3/4 vs. 0/4 successes (*p *= 0.0714); 4/4 vs. 0/4 successes (*p *= 0.0143); and 4/4 vs. 1/4 successes (*p *= 0.0714). For all other outcomes, *p *≥ 0.2143, and thus the study would be regarded as a “failure”.

**Table 5:** All possible outcomes, *p-*values, and corresponding decisions for an RCT with *n *= 8 patients (4 per treatment arm) with Fisher’s exact test

Number of responders		Difference in proportions (Experimental vs. Control)	Fisher’s exact test 1-sided *p-*value	Decision^a^
Experimental	**Control**			
0/4	0/4	0	1.0	**F**
1/4	1/4	0	0.7857	**F**
2/4	2/4	0	0.7571	**F**
3/4	3/4	0	0.7857	**F**
4/4	4/4	0	1.0	**F**
1/4	0/4	0.25	0.5	**F**
2/4	0/4	0.50	0.2143	**F**
3/4	0/4	0.75	0.0714	**S**
4/4	0/4	1	0.0143	**S**
0/4	1/4	-0.25	1.0	**F**
0/4	2/4	-0.50	1.0	**F**
0/4	3/4	-0.75	1.0	**F**
0/4	4/4	-1	1.0	**F**
2/4	1/4	0.25	0.5	**F**
3/4	1/4	0.50	0.2429	**F**
4/4	1/4	0.75	0.0714	**S**
1/4	2/4	-0.25	0.9286	**F**
1/4	3/4	-0.50	0.9857	**F**
1/4	4/4	-0.75	1.0	**F**
3/4	2/4	0.25	0.5	**F**
4/4	2/4	0.50	0.2143	**F**
2/4	3/4	-0.25	0.9286	**F**
2/4	4/4	-0.50	1.0	**F**
4/4	3/4	0.25	0.5	**F**
3/4	4/4	-0.25	1.0	**F**

^a^*F* Declare study a failure, *S *Declare study a success

Now let us consider a randomization-based inference approach. For illustration purposes, we consider four restricted randomization procedures—Rand, TBD, PBD(4), and PBD(2)—that exactly achieve 4:4 allocation. These procedures are legitimate choices because all of them provide exact sample sizes (4 per treatment group), which is essential in this trial. The reference set of either Rand or TBD includes $$70=\left(\begin{array}{c}8\\ 4\end{array}\right)$$ unique sequences though with different probabilities of observing each sequence. For Rand, these sequences are equiprobable, whereas for TBD, some sequences are more likely than others. For PBD($2b$), the size of the reference set is $${\left\{\left(\begin{array}{c}2b\\ b\end{array}\right)\right\}}^{B}$$, where $B=n/2b$ is the number of blocks of length $2b$ for a trial of size $n$ (in our example $n=8$). This results in in a reference set of ${2}^{4}=16$ unique sequences with equal probability of 1/16 for PBD(2), and of ${6}^{2}=36$ unique sequences with equal probability of 1/36 for PBD(4).

In practice, the study statistician picks a treatment sequence at random from the reference set according to the chosen design. The details (randomization seed, chosen sequence, etc.) are carefully documented and kept confidential. For the chosen sequence and the observed outcome data, a randomization-based *p-*value is the sum of probabilities of all sequences in the reference set that yield the result at least as large in favor of the experimental treatment as the one observed. This *p-*value will depend on the randomization design, the observed randomization sequence and the observed outcomes, and it may also be different from the population-based analysis *p-*value.

To illustrate this, suppose the chosen randomization sequence is CEECECCE (C stands for control and E stands for experimental), and the observed responses are FSSFFFFS (F stands for failure and S stands for success). Thus, we have 3/4 successes on experimental and 0/4 successes on control. Then, the randomization-based *p-*value is 0.0714 for Rand; 0.0469 for TBD, 0.1250 for PBD(2); 0.0833 for PBD(4); and it is 0.0714 for the population-based analysis. The coincidence of the randomization-based *p-*value for Rand and the *p-*value of the population-based analysis is not surprising. Fisher's exact test is a permutation test and in the case of Rand as randomization procedure, the *p-*value of a permutation test and of a randomization test are always equal. However, despite the numerical equality, we should be mindful of different assumptions (population/randomization model).

Likewise, randomization-based *p-*values can be derived for other combinations of observed randomization sequences and responses. All these details (the chosen randomization design, the analysis strategy, and corresponding decisions) would have to be fully specified upfront (before the trial starts) and agreed upon by both the sponsor and the regulator. This would remove any ambiguity when the trial data become available.

As the example shows, the level of evidence in the randomization-based inference approach depends on the chosen randomization procedure and the resulting decisions may be different depending on the specific procedure. For instance, if the level of significance is set to 10% as a criterion for a “successful trial”, then with the observed data (3/4 vs. 0/4), there would be a significant test result for TBD, Rand, PBD(4), but not for PBD(2).

## Conclusions

### Summary and discussion

Randomization is the foundation of any RCT involving treatment comparison. Randomization is not a single technique, but a very broad class of statistical methodologies for design and analysis of clinical trials [10]. In this paper, we focused on the randomized controlled two-arm trial designed with equal allocation, which is the gold standard research design to generate clinical evidence in support of regulatory submissions. Even in this relatively simple case, there are various restricted randomization procedures with different probabilistic structures and different statistical properties, and the choice of a randomization design for any RCT must be made judiciously.

For the 1:1 RCT, there is a dual goal of balancing treatment assignments while maintaining allocation randomness. Final balance in treatment totals frequently maximizes statistical power for treatment comparison. It is also important to maintain balance at intermediate steps during the trial, especially in long-term studies, to mitigate potential for chronological bias. At the same time, a procedure should have high degree of randomness so that treatment assignments within the sequence are not easily predictable; otherwise, the procedure may be vulnerable to selection bias, especially in open-label studies. While balance and randomness are competing criteria, it is possible to find restricted randomization procedures that provide a sensible tradeoff between these criteria, e.g. the MTI procedures, of which the big stick design (BSD) [37] with a suitably chosen MTI limit, such as BSD(3), has very appealing statistical properties. In practice, the choice of a randomization procedure should be made after a systematic evaluation of different candidate procedures under different experimental scenarios for the primary outcome, including cases when model assumptions are violated.

In our considered examples we showed that the choice of randomization design, data analytic technique (e.g. parametric or nonparametric model, with or without covariate adjustment), and the decision on whether to include randomization in the analysis (e.g. randomization-based or population model-based analysis) are all very important considerations. Furthermore, these examples highlight the importance of using randomization designs that provide strong encryption of the randomization sequence, importance of covariate adjustment in the analysis, and the value of statistical thinking in nonstandard RCTs with very small sample sizes and small patient horizon. Finally, in this paper we have discussed randomization-based tests as robust and valid alternatives to likelihood-based tests. Randomization-based inference is a useful approach in clinical trials and should be considered by clinical researchers more frequently [14].

### Further topics on randomization

Given the breadth of the subject of randomization, many important topics have been omitted from the current paper. Here we outline just a few of them.

In this paper, we have focused on the 1:1 RCT. However, clinical trials may involve more than two treatment arms. Extensions of equal randomization to the case of multiple treatment arms is relatively straightforward for many restricted randomization procedures [10]. Some trials with two or more treatment arms use unequal allocation (e.g. 2:1). Randomization procedures with unequal allocation ratios require careful consideration. For instance, an important and desirable feature is the allocation ratio preserving property (ARP). A randomization procedure targeting unequal allocation is said to be ARP, if at each allocation step the unconditional probability of a particular treatment assignment is the same as the target allocation proportion for this treatment [92]. Non-ARP procedures may have fluctuations in the unconditional randomization probability from allocation to allocation, which may be problematic [93]. Fortunately, some randomization procedures naturally possess the ARP property, and there are approaches to correct for a non-ARP deficiency – these should be considered in the design of RCTs with unequal allocation ratios [92–94].

In many RCTs, investigators may wish to prospectively balance treatment assignments with respect to important prognostic covariates. For a small number of categorical covariates one can use stratified randomization by applying separate MTI randomization procedures within strata [86]. However, a potential advantage of stratified randomization decreases as the number of stratification variables increases [95]. In trials where balance over a large number of covariates is sought and the sample size is small or moderate, one can consider covariate-adaptive randomization procedures that achieve balance within covariate margins, such as the minimization procedure [96, 97], optimal model-based procedures [46], or some other covariate-adaptive randomization technique [98]. To achieve valid and powerful results, covariate-adaptive randomization design must be followed by covariate-adjusted analysis [99]. Special considerations are required for covariate-adaptive randomization designs with more than two treatment arms and/or unequal allocation ratios [100].

In some clinical research settings, such as trials for rare and/or life threatening diseases, there is a strong ethical imperative to increase the chance of a trial participant to receive an empirically better treatment. Response-adaptive randomization (RAR) has been increasingly considered in practice, especially in oncology [101, 102]. Very extensive methodological research on RAR has been done [103, 104]. RAR is increasingly viewed as an important ingredient of complex clinical trials such as umbrella and platform trial designs [105, 106]. While RAR, when properly applied, has its merit, the topic has generated a lot of controversial discussions over the years [107–111]. Amid the ongoing COVID-19 pandemic, RCTs evaluating various experimental treatments for critically ill COVID-19 patients do incorporate RAR in their design; see, for example, the I-SPY COVID-19 trial (https://clinicaltrials.gov/ct2/show/NCT04488081).

Randomization can also be applied more broadly than in conventional RCT settings where randomization units are individual subjects. For instance, in a cluster randomized trial, not individuals but groups of individuals (clusters) are randomized among one or more interventions or the control [112]. Observations from individuals within a given cluster cannot be regarded as independent, and special statistical techniques are required to design and analyze cluster-randomized experiments. In some clinical trial designs, randomization is applied within subjects. For instance, the micro-randomized trial (MRT) is a novel design for development of mobile treatment interventions in which randomization is applied to select different treatment options for individual participants over time to optimally support individuals’ health behaviors [113].

Finally, beyond the scope of the present paper are the regulatory perspectives on randomization and practical implementation aspects, including statistical software and information systems to generate randomization schedules in real time. We hope to cover these topics in subsequent papers.

## Supplementary Information

**Additional file 1: Figure S1**. Type I error rate under selection bias model with bias effect ($\nu$) in the range 0 (no bias) to 1 (strong bias) for 12 randomization designs and three statistical tests.

## Data Availability

All results reported in this paper are based either on theoretical considerations or simulation evidence. The computer code (using R and Julia programming languages) is fully documented and is available upon reasonable request.

## Footnotes

1 Guess the next allocation as the treatment with fewest allocations in the sequence thus far, or make a random guess if the treatment numbers are equal.
