# Supplementary material for: A roadmap to using randomization in clinical trials
Source: BMC Med Res Methodol. 2021 Aug 16;21:168. doi: 10.1186/s12874-021-01303-z (PMC8366748; doi:10.1186/s12874-021-01303-z)

# Supplementary Material

This document contains additional simulation output for the manuscript “A roadmap to using randomization in clinical trials”

### Figure S1: Type I error rate under selection bias model with bias effect ($\nu$) in the range 0 (no bias) to 1 (strong bias) for 12 randomization designs and three statistical tests.


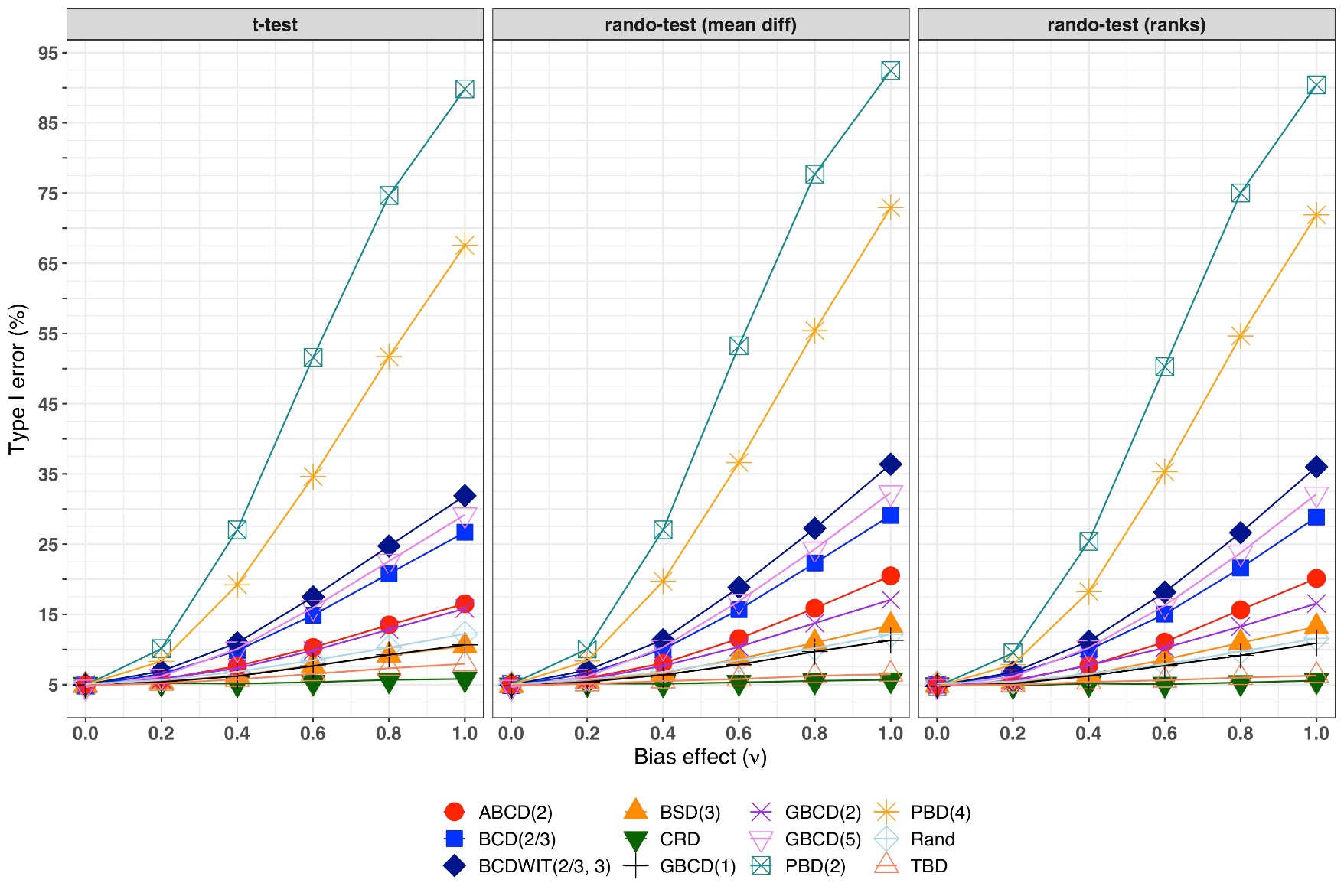

Supplement: Supplementary file 1 — Additional file 1: Figure S1. Type I error rate under selection bias model with bias effect ($\nu$ν) in the range 0 (no bias) to 1 (strong bias) for 12 randomization designs and three statistical tests. [file 12874_2021_1303_MOESM1_ESM.docx]
